# Cetuximab Plus Platinum-Based Chemotherapy in Head and Neck Squamous Cell Carcinoma: A Retrospective Study in a Single Comprehensive European Cancer Institution

**DOI:** 10.1371/journal.pone.0086697

**Published:** 2014-02-06

**Authors:** Ramon Andrade de Mello, Sandra Gerós, Marcos Pantarotto Alves, Filipa Moreira, Isabel Avezedo, José Dinis

**Affiliations:** 1 Department of Medical Oncology, Head and Neck Unit, Portuguese Oncology Institute, Porto, Portugal; 2 Department of Medicine and Biomedical Sciences, School of Medicine, University of Algarve, Faro, Portugal; 3 Department of Medicine, Faculty of Medicine, University of Porto, Porto, Portugal; 4 Service of Otorhinolaryngology, Head and Neck Unit, Portuguese Oncology Institute, Porto, Portugal; 5 Department of Otorhinolaryngology and Cervical Facial Surgery, Centro Hospitalar de Vila Nova de Gaia/Espinho, Vila Nova de Gaia, Portugal; University of Nebraska Medical Center, United States of America

## Abstract

**Background:**

The use of cetuximab in combination with platinum (P) plus 5-fluorouracil (F) has previously been demonstrated to be effective in the treatment of metastatic squamous cell cancer of head and neck (SCCHN). We investigated the efficacy and outcome of this protocol as a first-line treatment for patients with recurrent or metastatic disease. We evaluated overall-survival (OS), progression-free-survival (PFS), overall response rate (ORR) and the treatment toxicity profile in a retrospective cohort.

**Patients and Methods:**

This study enrolled 121 patients with untreated recurrent or metastatic SCCHN. The patients received PF+ cetuximab every 3 weeks for a maximum of 6 cycles. Patients with stable disease who received PF+ cetuximab continued to receive cetuximab until disease progressed or unacceptable toxic effects were experienced, whichever occurred first.

**Results:**

The median patient age was 53 (37–78) years. The patient cohort was 86.8% male. The addition of cetuximab to PF in the recurrent or metastatic setting provided an OS of 11 months (Confidential Interval, CI, 95%, 8.684–13.316) and PFS of 8 months (CI 95%, 6.051–9.949). The disease control rate was 48.9%, and the ORR was 23.91%. The most common grade 3 or 4 adverse events in the PF+ cetuximab regimen were febrile neutropenia (5.7%), skin rash (3.8%) and mucosistis (3.8%).

**Conclusions:**

The results of this study suggest that cetuximab plus platinum–fluorouracil chemotherapy is a good option for systemic treatment in advanced SSCHN patients. This regimen has a well-tolerated toxicity profile.

## Introduction

Squamous cell carcinoma of the head and neck (SCCHN), including the oral cavity, nasopharynx, hypopharynx, larynx and tongue, is the 5^th^ most common cancer worldwide and represents 4% of all diagnosed neoplasms [1]. The annual world incidence is approximately 500,000 new cases, and patients with metastatic disease have very poor outcomes [2]. In Europe, head and neck tumors account for 139,000 new cases per year [3], [4]. Currently, patients with operable and early-stage disease receive conservative surgery or radiotherapy as the standard of care. Induction chemotherapy with TPF (taxane, platinum and fluoropirimidine) followed by radiotherapy or chemo-radiotherapy is an option for organ preservation in advanced larynx and hypopharynx patients otherwise requiring laryngectomy [5]. In the recurrent/metastatic disease setting, the 5-year OS rate is approximately 39.4% [6]. However, the survival among patients with head and neck cancer has only modestly improved over the past 30 years [7]. Many international centers advocate salvage surgery as the primary option for recurrent SCCHN [7]. However, for patients ineligible for surgery, platinum-based chemotherapy is the backbone of treatment [5], [8], [9]. Many trials have accessed doublet [2], [8], [10] and triplet drug [8], [11]–[13] combinations in the recurrent/metastatic setting and have shown modest outcomes. Epidermal growth factor receptor (EGFR) pathways were shown in previous pre-clinical studies to have a major role in SCCHN carcinogenesis by regulating p53 and Rb gene expression. p53 and Rb are regulators of cell cycle control, cell proliferation and apoptosis [14], [15]. More recently, cetuximab, an IgG1 monoclonal antibody against the extracellular portion of the epidermal growth factor receptor (EGFR), was extensively studied in this field [9], [13], [16]. Since 2005, several phase I-III trials [8], [11]–[13] have assessed cetuximab in combination with standard chemotherapy for the treat of recurrent/metastatic SCCHN. In 2008, Vermoken *et al.* [3] published the results of an interesting phase III trial that showed improved outcome results with a combination of cetuximab with platinum plus 5-fluourouracil (5-FU) in the treatment of advanced SCCHN patients. These results led to the approval of this regimen in Europe and the United States. Herein, we report the results of a retrospective study with the primary end-point of assessing outcomes in a southern European comprehensive cancer institution. We analyzed overall survival (OS) and progression-free-survival (PFS) after the addition of cetuxumab to a platinum plus 5-FU regimen. The secondary end-points of this study were the assessment of treatment related-toxicities and disease control.

## Patients and Methods

### Design

Our study was conducted from January 2010 to January 2013 at the Central Comprehensive Cancer Hospital in northern Portugal: the Portuguese Oncology Institute (IPO-PORTO), Porto, Portugal. The study was approved by the IPO-PORTO's ethical committee and was conducted according to the Declaration of Helsinki. Signed written-informed consent form was obtained from all patients involved in this study.

### Patients

The patient inclusion criteria were the following: confirmed histologic diagnosis of recurrent/metastatic squamous cell carcinoma of head and neck, age greater than 18 years, ineligibility for local therapy, at least one lesion that was bi-dimensionally measurable by computed tomography (CT), an Eastern Cooperative Oncology Group (ECOG) performance status score of 0–2, and adequate hematologic, renal, and hepatic function. No tumor tissue was assessed for *EGFR* or for human papilloma virus (HPV) expression. Patients who did not meet the inclusion criteria were excluded from this study. Other exclusion criteria were surgery or irradiation within the previous 4 weeks, previous systemic chemotherapy unless it was part of multimodal treatment for locally advanced disease that had been completed more than 6 months before study entry, nasopharyngeal carcinoma and other concomitant anticancer therapies. Data were collected from clinical records at the participant institution. All patients involved in this study were Portuguese Caucasians.

### Treatment schedule regimen

Selected patients were submitted to systemic treatment with either cisplatin (at a dose of 100 mg/m^2^ body-surface area as a 2-hour intravenous infusion on day 1) or carboplatin (at an area under the curve of 5 mg per milliliter per minute, as a 1-hour intravenous infusion on day 1). The patients received an infusion of fluorouracil (at a dose of 1000 mg/m^2^ per day for 4 days under continuous infusion) every 3 weeks for six cycles. The use of cisplatin or carboplatin was determined according to patient fitness status and physician discretion. Cetuximab was administered at an initial dose of 400 mg/m^2^ given as a 2-hour intravenous infusion, followed by subsequent weekly doses of 250 mg/m^2^ given as a 1-hour intravenous infusion. The cetuximab infusions ended at least 1 hour before the start of chemotherapy. After a maximum of six cycles of chemotherapy, patients who had at least stable disease received cetuximab monotherapy until disease progression or unacceptable toxicity.

### End-points

The primary end-point of our study was overall survival, which was defined as the period between the date of death/last medical visit and date of first recurrence/metastasis diagnosis. Progression-free-survival was defined as the period between the date of second recurrence/metastasis diagnosis and the date of first recurrence/metastasis diagnosis. The secondary end-point was overall response rate (ORR), which included complete response (CR) and partial response (PR). Responses were defined according to Response Evaluation Criteria in Solid Tumors (RECIST) [17]. Disease control (ORR+ stable disease) and toxicity profiles were extracted from clinical records according to the Common Terminology Criteria for Adverse Events version 4.0. Patients were considered evaluable for efficacy if they completed at least 3 cycles of treatment per institution protocol analysis. Tumors were assessed by CT scan at baseline and after 3 cycles of combination therapy (approximately 12 weeks from the start of therapy). If a patient did not tolerate the treatment due to grade 3 and 4 toxicities, the main approach was to stop the treatment and select a second-line treatment if the patient was fit to receive another treatment. If patients progressed quickly, the treatment was stopped and the patients were offered alternate treatment options.

### Statistical analysis

The chi-squared and Wilcoxon-Mann Whitney tests were used to compare the frequency distributions of variables such as age, sex, tumor site, extent of disease, ECOG performance status, smoking status, histologic type, previous treatment, and Tumor, Node, Metastasis (TNM) stage classification in the study population. We analyzed OS and PFS using a Kaplan-Meier curve. All statistical tests were two-sided, and *p*<0.05 was considered the threshold of statistical significance. All data analyses were performed using IBM® SPSS Statistics, version 21.0 (Chicago, USA).

## Results

### Patient characteristics

Table 1 summarizes the characteristics of the 121 patients involved in our study. The majority of the study participants were male (86.8%). The median patient age was 53 (37–78) years, and 90.9% of the patients were less than 65 years old. Major risk factors such as tobacco and high daily alcohol consumption were also observed. The primary tumor sites were the oropharynx (18.3%), hypopharynx (20.8%), larynx (25%) and oral cavity (31.7%). Locally regional tumors were predominant (52.3%), and histologic types were well differentiated (32.5%), moderately differentiated (35%), and poorly differentiated (32.5%). Cisplatin was the preferred platinum therapy used (57.8%).

**Table 1:** Characteristics of patients treated with platinum in combination with 5-FU plus cetuximab.

	No.	%
**No. of patients**	121	
**Male**	105	86.8
**Age (years)**	53 (37–78)	
**<65 years**	110	90.9
**≥65 years**	11	9.1
**ECOG PS**		
**0**	4/103	3.9
**1**	83/103	80.6
**2**	13/103	12.6
**3**	3/103	2.9
**4**	0	0
**Missing data**	18	-
**Tobacco use**		
**Smoker**	70/91	76.1
**Ex-smoker**	10/91	10.9
**Never-smoker**	11/91	12
**Missing data**	30	-
**Alcohol**		
**≥60 g/day**	74/86	86.04
**<60 g/day**	12/86	13.95
**Missing data**	35	
**Primary tumor site**		
**Oropharynx**	22/120	18.3
**Hypopharynx**	25/120	20.8
**Larynx**	30/120	25
**Oral Cavity**	38/120	31.7
**Others**	5/120	4.2
**Missing data**	1	-
**Extent of diseasse**		
**Only locoregionally**	56/106	52.8
**Metastatic/local recurrence**	50/106	47.2
**Missing data**	15	-
**Histologic grade**		
**Well differentiated**	13/40	32.5
**Moderately differentiated**	14/40	35
**Poorly differentiated**	13/40	32.5
**Not specified**	11	-
**Missing data**	70	-
**Platinum**		
**Cisplatin**	63/109	57.8
**Carboplatin**	46/109	42.2
**Missing data**	12	-
**Previous treatment**		
**Chemotherapy**	28/108	25.9
**Radiotherapy**	25/108	23.1
**Chemo-radiotherapy**	35/108	32.4
**Missing data**	13	-
**CENSOR**		
**No. deaths**	88	86.3

**Abbreviations**: ECOG PS, eastern cooperative oncology group performance status; 5-FU, 5-fluourouracil.

### Treatment response rates

Table 2 and Table 3 summarize data on the ORRs assessed after 3 and 6 cycles of platinum, 5-FU and cetuximab and cetuximab maintenance treatment. Furthermore, Table 3 shows the specific ORR among platinum options used in the protocol: cisplatin versus carboplatin. The median duration of disease control was 11 (0–115) weeks and accounted for 48.91% of patients assessed. A complete response was observed in 6 patients (6.5%), and a partial response was observed in 16 patients (17.4%). Stable disease was observed in 23 patients (25%), and disease progression occurred in 47 patients (51.08%). The median duration of maintenance treatment with cetuximab was 17 (0–85) weeks. There were no statistical differences found for ORR with respect to the platinum option used for the PF+ cetuximab protocol. However, a trend towards improved outcomes in the cisplatin group was noted (Table 3).

**Table 2:** Treatment characteristics after 3 or 6 cycles of platinum, 5-fluourouracil and cetuximab.

	No.	%
**Patients enrolled**	121	-
**PF+ cetuximab**		
**Complete response**	6/92	6.52
**Partial response**	16/92	17.39
**Stable disease**	23/92	25
**Progression or without response**	47/92	51.08
**Missing data** +	29	-
**Overall response rate (ORR)** *	22/92	23.91
**Disease control** **	45/92	48.91
**Duration of ORR (weeks)** ***	11 (0–115)	
**Cetuximab maintenance**		
**No response**	38/45	84.4
**Stable disease**	7/45	15.6
**Without maintenance**	47	-
**Missing data**	29	-
**Duration of maintenance (weeks)**	17 (0–85)	

**Abbreviations**: PF, platinum (cisplatin or carboplatin) and 5-fluourouracil.

+ Include patients who have not image assessment before cycle 3 due to toxicities issues.

* ORR refers to overall response rate and includes complete response and partial response, according to RECIST criteria.

*** ORR+stable disease.

** Median.

**Table 3:** Treatment characteristics according to platinum used for CF+ cetuximab.

	Cisplatin (%)	Carboplatin (%)	*p* value
**Patients enrolled**	53 (57.6)	39 (42.4)	
**Age (years)** **	53 (37–78)	57 (37–75)	0.024***
**PF+ cetuximab**			
**Complete response**	3/53 (5.7)	3/39 (7.7)	
**Partial response**	13/53 (24.5)	3/39 (7.7)	0.216*
**Stable disease**	12/53 (22.6)	11/39 (28.2)	
**Progression or without response**	25/53 (47.2)	22/39 (56.4)	
**ORR** +	16/53 (30.2)	6/39 (15.4)	0.258*
**Disease control rate** ++	28/53 (52.8)	17/39 (43.6)	0.381*
**Duration of ORR (weeks)** **	11.5 (0–115)	11 (0–91)	0.427***
**Cetuximab maintenance**			
**No response**	24/26 (92.3)	14/19 (73.7)	
**Stable disease**	2/26 (7.7)	5/19 (26.3)	0.089*
**Duration of maintenance (weeks)** **	18 (0–58)	15 (3–85)	0.780***

**Abbreviations**: PF, platinum (cisplatin or carboplatin) and 5-fluourouracil.

+ ORR refers to overall response rate and includes complete response and partial response.

++ Disease control rate includes complete response, partial response and stable disease.

* Qui square test.

** Median.

*** Mann-Whitney U Test.

### Safety and tolerability

The worst grade 3 and grade 4 adverse events (AEs) for patients who were treated with the PF+ cetuximab protocol and for patients who received cetuximab maintenance treatment are reported in Table 4. Among the patients treated with the cisplatin/5FU+ cetuximab regimen, the most commonly reported AEs were febrile neutropenia (6.8%), neutropenia (6.8%), hypomagnesemia (3.4%), mucositis (1.7%) and pneumonia (1.7%). Among patients treated with the carboplatin/5FU+ cetuximab regimen, the most commonly reported AEs were skin rash (8.7%), mucositis (6.5%), febrile neutropenia (4.3%), pneumonia (4.3%), anemia (3.5%) and hypomagnesemia (2.2%). With respect to cetuximab maintenance, skin rash was noteworthy (6.4%) among patients treated in our cohort.

**Table 4:** Grade 3 and/or grade 4 adverse effects observed according to CTCAE version 4.0.

	PF+ cetuximab		Cisplatin +5FU+cetuximab		Carbooplatin +5FU+cetuximab		Cetuximab	
	No.	%	No.	%	No.	%	No.	%
**Febrile**	6	5.7	4	6.8	2	4.3	1	0.9
**Neutropenia**	5	4.7	4	6.8	1	2.2	0	-
**Skin rash**	4	3.8	0	-	4	8.7	7	6.4
**Mucositis**	4	3.8	1	1.7	3	6.5	1	0.9
**Anemia**	3	2.8	0	-	3	3.5	0	-
**Hypomagnesemia**	3	2.8	2	3.4	1	2.2	0	-
**Pneumonia**	3	2.8	1	1.7	2	4.3	0	-
**Dispneia**	0	-	0	-	0	-	1	0.9
**Sepsis**	1	0.9	1	1.7	0	-	0	-
**Infusion reactions**	1	0.9	1	1.7	0	-	0	-
**Vomiting**	1	0.9	1	1.7	0	-	0	-
**Low platelet count**	1	0.9	1	1.7	0	-	0	-

**Abbreviations**: CTCAE, Common Terminology Criteria for Adverse Events. 5FU, 5-fluourouracil.

### Outcomes: progression-free-survival and overall survival

**Figure 1:**
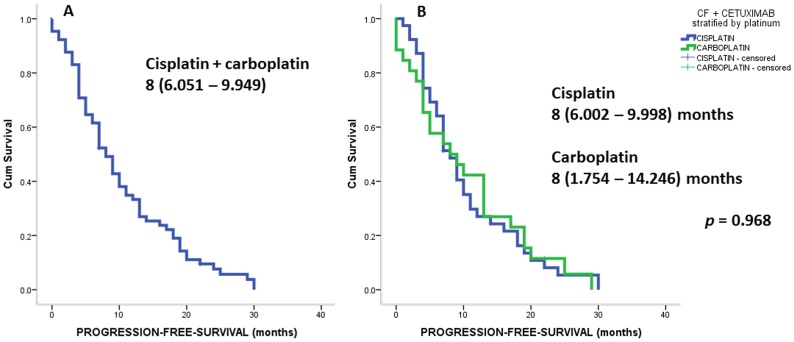
Show PFS of all patient treated with platinum +5-FU +cetuximab (A); and stratified by platinum (B): carboplatin +5-FU+cetuximab versus cisplatin +5-FU+cetuximab. Analysis was performed using log-rank test. Abbreviations: PFS stands for progression-free-survival; C stands for platinum (carboplatin or cisplatin); 5-FU stands for 5-fluourouracil.

**Figure 2:**
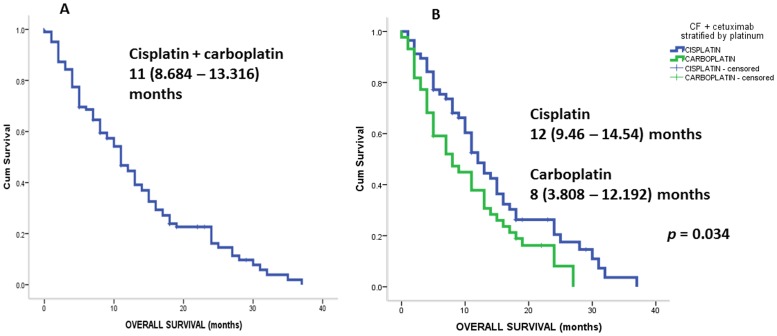
Show OS survival of all patients treated with platinum +5-FU+cetuximab (A); and stratified by platinum (B): carboplatin +5-FU+cetuximab versus cisplatin +5-FU+cetuximab. Analysis was performed using log-rank test. Abbreviations: OS stands for overall-survival; C stands for platinum (carboplatin or cisplatin); 5-FU stands for 5-fluourouracil.

Figure 1 and Figure 2 show the outcomes for PFS and OS. The mortality rate was 86.3% during this retrospective cohort assessment. The median follow-up period was 24 months. Figure 1A shows PFS of all 121 patients involved in this study, including those treated both with cisplatin, 5-FU, cetuximab or carboplatin, 5-FU and cetuximab. The PFS was 8 months (95% confidential interval (CI), 6.051–9.949). Figure 1B provides data regarding platinum stratification sub-group PFS (cisplatin versus carboplatin): 8 (95%CI, 6.002–9.998) versus 8 (95% CI, 1.754–14.246) months, *p* = 0.968. Figure 2A shows the OS of all patients treated with platinum (cisplatin or carboplatin), 5-FU and cetuximab. The OS was 11 months (95%CI, 8.684–13.316). In addition to these results, Figure 2B shows the OS of sub-groups stratified by platinum treatment (cisplatin versus carboplatin). The OS was 12 months for cisplatin (9.460–14.540) versus 8 months (3.808–12.192) for carbolatin, *p* = 0.034.

## Discussion

The treatment of advanced SCCHN is still a challenge for surgeons, radio-oncologists and medical oncologists worldwide. A multidisciplinary schedule should be established in all cases to provide optimized approaches [4]. In recurrent and/or metastatic disease, systemic treatments have had a major role in improving survival and quality of life [16]. In 2008, a major advance in SCCHC treatment was provided with the addition of cetuximab to platinum and 5-FU chemotherapy [3]. Other trials have attempted to assess alternative choices for controlling metastatic disease, such as erlotinib, lapatinib, afatinib, rilotumumab, ficlatuziumab and ornatuzumab, but the data are preliminary [18]–[20]. This retrospective study was very important because it assessed the role of cetuximab in association with platinum-fluoropirimide chemotherapy for SCCHN in a southern European comprehensive cancer institution. In this study, 121 patients treated with this regimen were assessed, and the outcomes were similar to those of the EXTREME trial conducted by Vermoken *et al.* [3] that led to the approval of this regimen. Vermoken's study assessed 222 recurrent/advanced SCCHN patients who underwent screening at 81 centers in 17 European countries. Cisplatin was administered as the initial platinum-based treatment in 149 (67%) patients. The median OS was 10.1 (95% CI, 8.6–11.2) months, and the median PFS was 5.6 (95% CI, 5.0–6.0) months. The ORR was 36% in that patient group. Although our retrospective study was performed in only one European center, the sample size was large (121 patients) and included approximately half of the total number of patients involved in the EXTREME trial. Thus, the analysis described herein provides valuable data regarding what actually occurs outside of a clinical trial. The results were quite similar to those first presented in the literature despite a small trend toward an improved disease control rate (48.9%), PFS (8 months) and OS (11 months). This result may be explained by ethnic differences among the study populations. Our study used a predominantly Portuguese population, and a heterogeneous European population was involved in the EXTREME trial [15], [21]–[24]. In addition, SCCHN tumors are rich in EGFR, which may explain the high sensitivity to anti-EGFR therapies [25]. Previous studies have shown that epidermal growth factor (*EGF*) +61 A/G polymorphisms are associated with cancer susceptibility and EGF tumor expression [26]. In Portugal, several studies have assessed the role of epidermal growth factor and its receptor regulation with respect to cancer susceptibility for gliomas [27], gastric cancer [28] and lung cancer [22]. Therefore, we hypothesize that tumors of epithelial origin exhibit high EGF expression in the Portuguese population and that these tumors are more sensitive to anti-EGFR agents such as cetuximab. However, further studies assessing EGFR expression in tumor tissue should be performed to validate our hypothesis. In addition, the majority of patients (57.8%) received cisplatin-based regimens that were associated with improved OS compared to carboplatin-based regimens: 12 (95%, CI, 9.46–14.54) versus 8 (95%, CI, 3.308–12.192) months, *p* = 0.034. The results of this study also confirm the superior sensitivity of cisplatin-based regimens in association with cetuximab compared to carboplatin-based regimens that were previously reported in the literature [9]. Carboplatin-based regimens are reserved for patients who may not tolerate cisplatin because of poor ECOG status or other co-morbidities, such as diabetes (with neuropathy) or previous stage I–III renal failure [29]. In addition to these results, the toxicity profile presented was very acceptable and controlled among patients treated in this cohort. The rate of febrile neutropenia was lower than that reported in the EXTREME trial for PF+ cetuximab (5.7% versus 22%). This result may be explained by the previous treatment with routine prophylactic antibiotic-therapy with ciprofloxacin and G-CSF (granulocytic and colony stimulate factor) that the patients received [30]–[32]. The patients experienced fewer grade 3 or grade 4 AEs than the patients involved in the EXTREME trial [3]: skin rash, 3.8 versus 9%; anemia, 2.8% versus 13%; thrombocytopenia, 0.9 versus 11%; hypomagnesemia, 2.8 versus 5%; pneumonia, 2.8% versus 4%; sepsis, 0.9% versus 4%; vomiting, 0.9% versus 5%, respectively (table 4). However, the patients experienced more mucositis grade 3 or 4 events (3.8%) than previously reported for the PF+ cetuximab regimen [3]. The regimen toxicity profile could be more uniform and could be better managed in a single European comprehensive institution study than in a multi-center study that involved 81 centers in 17 different European countries. The chemotherapy supportive care to control emesis [33]–[35], hematologic effects [30], [36]–[38] and infections [30], [32] would depend on different local protocols, populations, environmental exposures and public health conditions. For patients with recurrent/metastatic disease and low but adequate fitness performance status, other options such as cetuximab and placlitaxel [10], cetuximab and bevacizumab [39], oxaliplatin, infusional-5-FU and cetuximab [12] have been studied and showed promising results. In 2012, Hitt *et al.* [10] published the results of a phase II study that assessed 46 advanced SCCHN patients who received paclitaxel 80 mg/m^2^ and cetuximab 400/250 mg/m^2^ weekly until disease progression or unacceptable toxicity. The ORR was 54% (95% CI, 39–69%), and the PFS was 4.2 (95% CI, 2.9–5.5) months. The OS was shown to be 8.1 (95% CI, 6.6–9.6) months. The most common grade 3 or 4 AEs were skin rash (24%), asthenia (17%) and neutropenia (13%). The authors concluded that this regimen is safe and well-tolerated and had promising outcomes for medically unfit patients and patients for whom platinum is contraindicated. In 2013, Argiris *et al.* [39] reported the results of another phase II trial enrolling 46 advanced SCCHN patients who received weekly cetuximab 400/250 mg/m^2^ and bevacizumab 15 mg/m^2^ on day 1 given intravenously every 21 days until disease progression or the occurrence of unacceptable AEs. The ORR was 16%, the PFS was 2.8 months, and the OS was 7.5 months. The most common grade 3 or 4 AEs occurred in less than 10% of all patients. Despite these modest results, several phase III studies are still required to determine the role of biological agent combinations in this patient population.

## Conclusions

The combination of anti-EGFR therapies with platinum-based chemotherapy is a cornerstone in the new era of SCCHN treatment. Over the last 30 years, there have been no significant innovations concerning systemic treatment for recurrent/metastatic disease. Cetuximab has emerged as a key player in the treatment of SCCHN patients in association with platinum and 5-FU. To the best of our knowledge, our retrospective study is the first to report on the medical experience of this regimen in a relatively large southern European Portuguese population. Furthermore, we confirmed that the results were in agreement with the literature. Thus, the cisplatin-based PF+ cetuximab regimen is a good option for systemic treatment in medically fit advanced SSCHN patients. Moreover, the treatment has a well-tolerated toxicity profile. Further studies are warranted to determine biomarkers for personalizing therapies and improving outcomes in this set of patients.
